# Supplementary material for: Hepato‐entrained B220+ CD11c+ NK1.1+ cells regulate pre‐metastatic niche formation in the lung
Source: EMBO Mol Med. 2018 Jun 21;10(7):e8643. doi: 10.15252/emmm.201708643 (PMC6034134; doi:10.15252/emmm.201708643)
Supplement: Supplementary file 1 — Appendix [file EMMM-10-e8643-s001.pdf]

Appendix: Hepato-entrained B220$^+$CD11c$^+$NK1.1$^+$ cells regulate pre-metastatic niche formation in the lung

Table of Contents:

Appendix Figure S1. BM-derived CD45$^+$ cells showed FX expression in liver in tumour-bearing mice.

Appendix Figure S2. Representative immunohistochemical images of fibrinogen deposition in no tumour-bearing- and tumour-bearing- mouse lungs.

Appendix Figure S3. Flow cytmetric analyses of spleen cells.

Appendix Figure S4. Flow cytmetric analyses of lung cells derived from KikGR mice that blood perfusion were carried out.

Appendix Figure S5. Induction of HepEL relocation in TCM-stimulated lungs.

Appendix Figure S6. Gating strategy for lung cells that contained the relocated HepELs, which were photoconverted in liver in a TCM-stimulated KikGR mouse.

Appendix Figure S7. Tracing of B220$^+$CD11c$^+$NK1.1$^+$ cells in various organs such as lung, liver, bone marrow, peripheral blood, lymph node and primary tumours during the progression of tumour.

Appendix Figure S8. Quantification of fibrinogen deposition area in tumor-bearing wild-type (WT) and FX$^{-/-}$ mouse lungs.

Appendix Figure S9. Metastatic tumor cells in tumor-bearing FX-/- mouse lungs that were treated with wild-type, FX+/- or FX-/- HepELs.

Appendix Table S1. A list of the top 50 genes that were significantly up-regulated in peripheral leukocytes in LLC-bearing or B16-bearing mice.

Appendix Figure S2. Gene expression in photoconverted HepELs derived from TCM-stimulated- lungs and livers.

Appendix Fig. S1

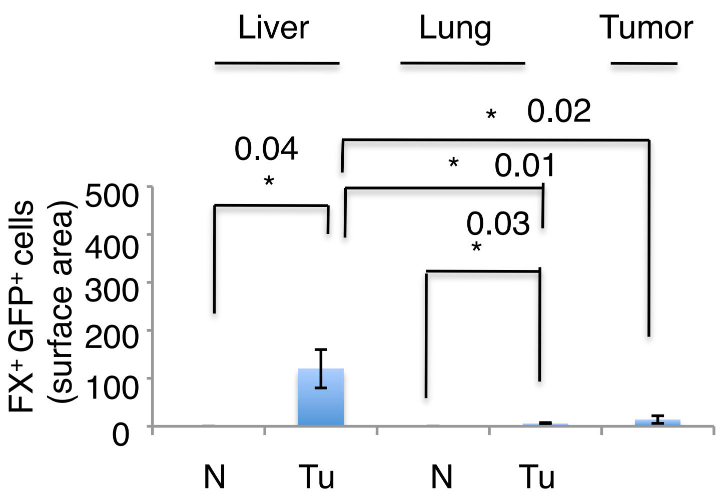

N : no tumor-bearing
Tu : tumour-bearing

BM-derived CD45⁺ cells showed FX expression in liver in tumour-bearing mice
IHC examination was carried out in tumour-bearing- lung, liver and primary tumour (tumour diameter: 7 mm) that were derived from bone marrow transplantation (GFP⁺-BM) mice. N = 5.

Appendix Fig. S2

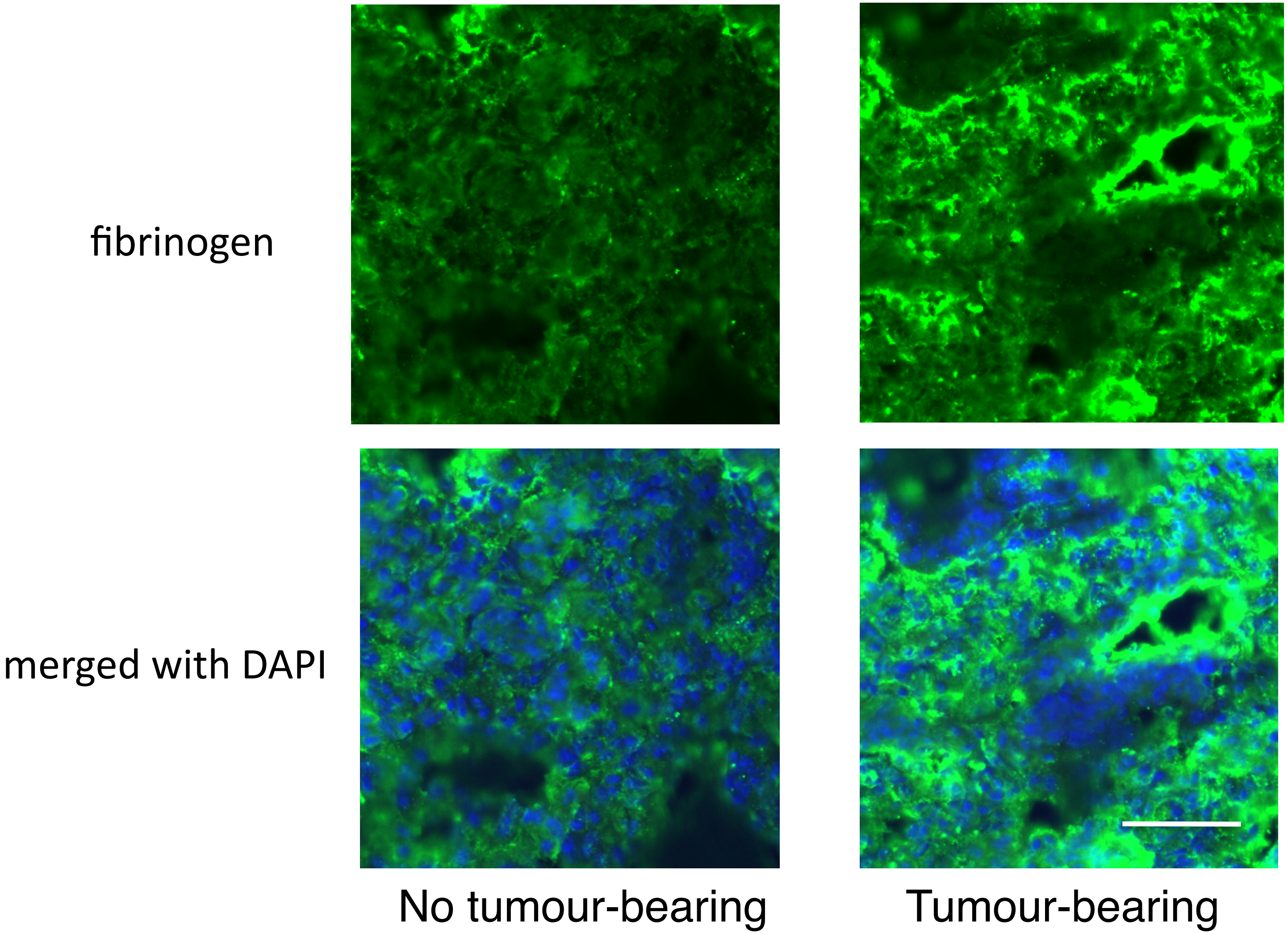

Representative immunohistochemical images of fibrinogen deposition in no tumour-bearing- and tumour-bearing-mouse lungs (scale bar, 50 μm).

Appendix Fig. S3

Spleen

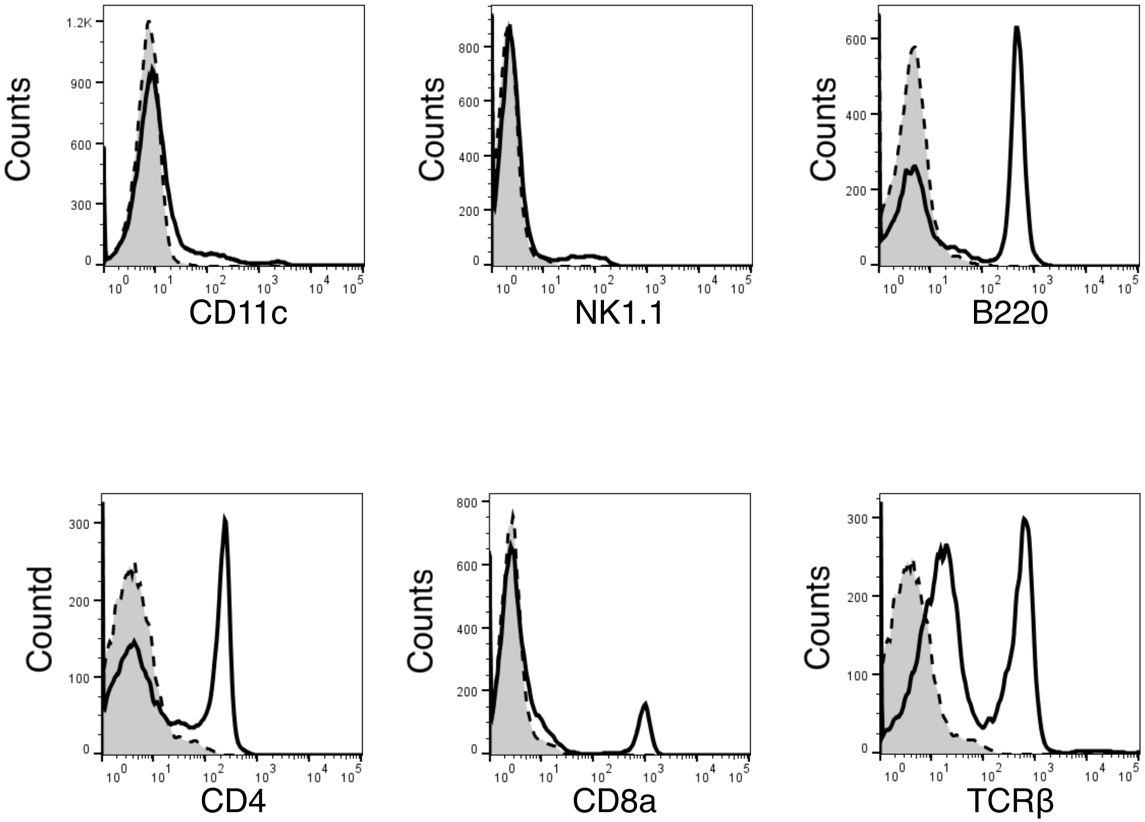

Flow cytmetric analyses of spleen cells. Histograms of isotype control-Ab (dashed line) and specific-Ab (bold line) stained cells are depicted. The flow cytmetric analyses in Fig. EV1 and Appendix Fig. 3-4 were carried out based on this staining. Some of Abs such as B220, CD4 and CD8 detected bright signals in spleen cells compared with lung cells.

Appendix Fig. S4

lung

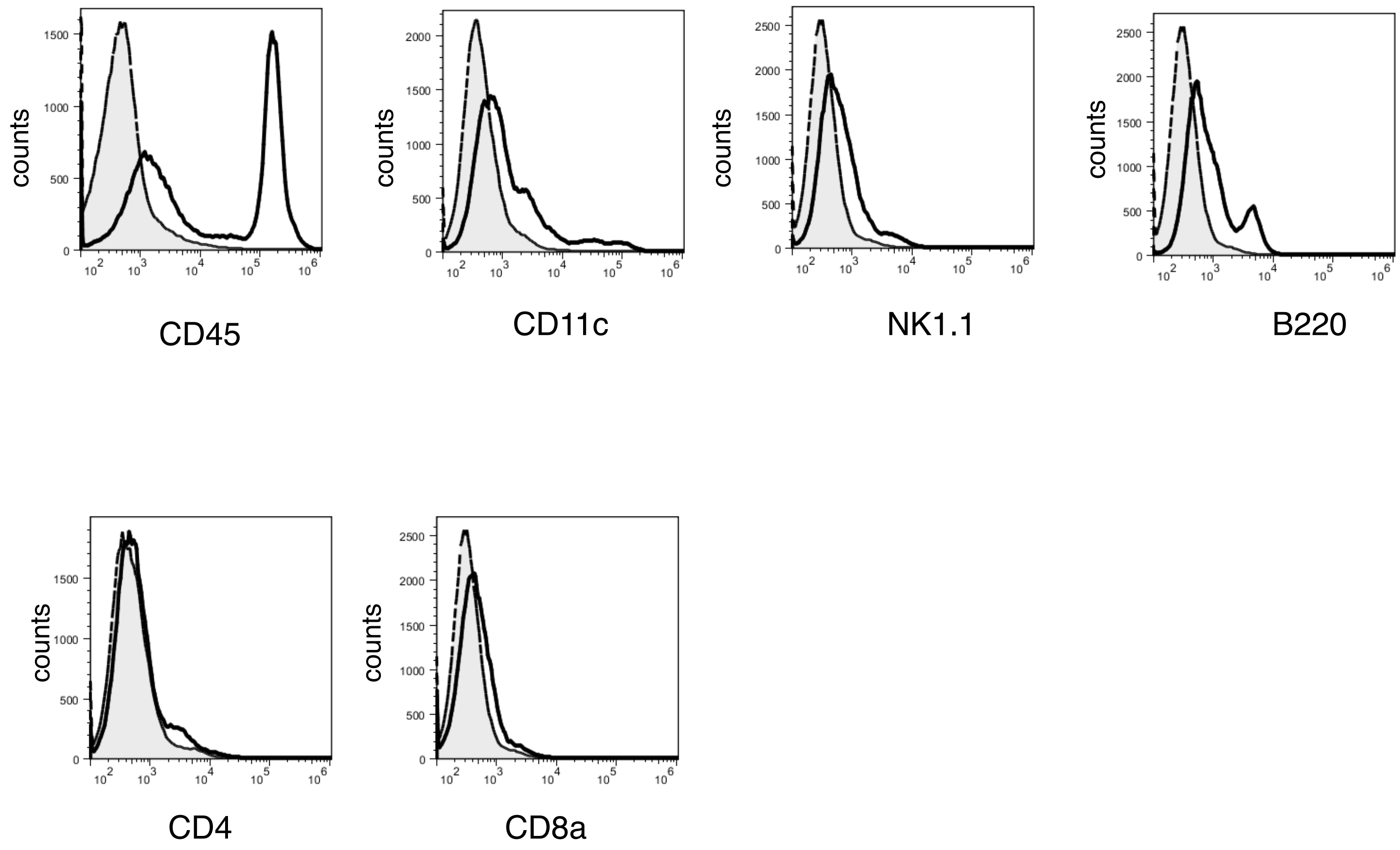

Flow cytmetric analyses of lung cells derived from KikGR mice that blood perfusion were carried out. Histograms of isotype control-Ab (dashed line) and specific-Ab (bold line) stained cells are depicted. The flow cytmetric analyses in Fig. EV1 and Appendix Fig. 3-4 were carried out based on this staining.

Appendix Fig. S5

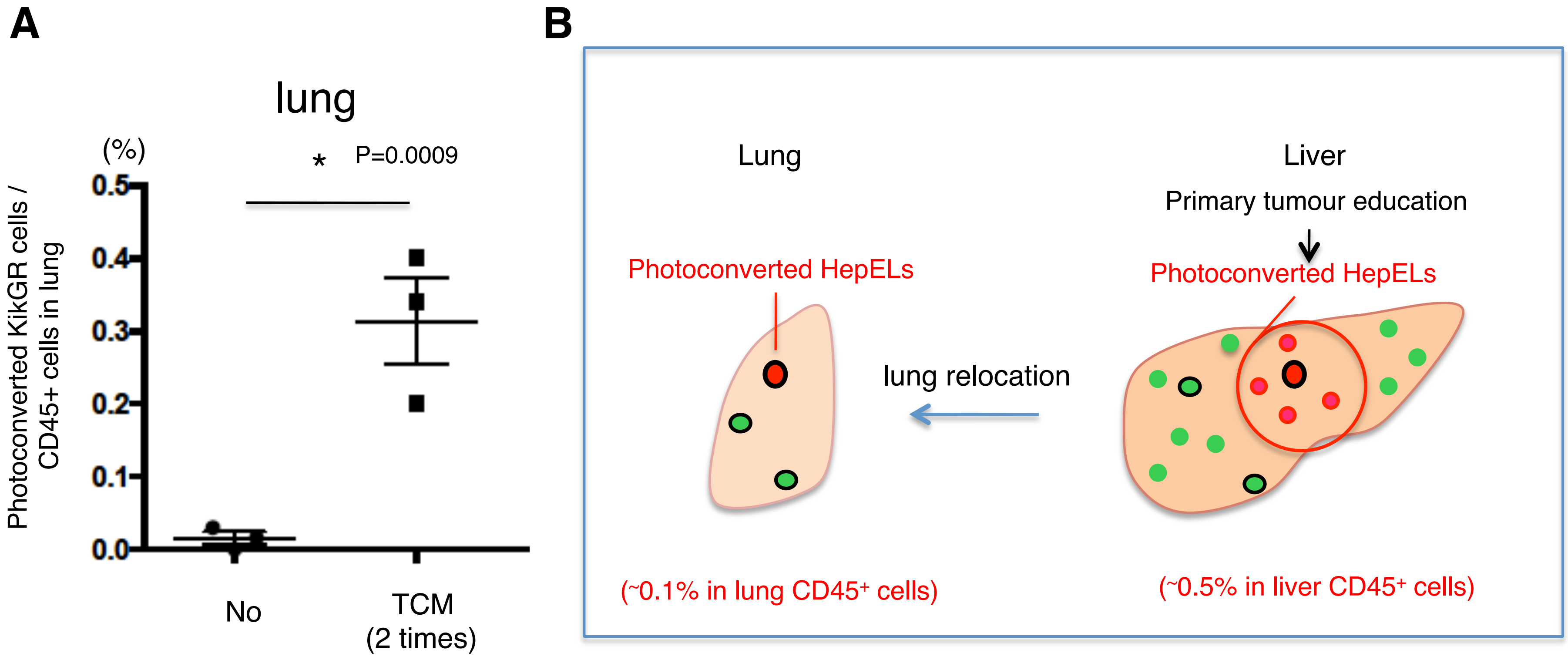

Induction of HepEL relocation in TCM-stimulated lungs. (**A**) Flow cytometric quantifications of the photoconverted (KikGR red) cells in lungs. No: no stimulation. N=3 (littermate), (No, TCM (2 times, every 2 days)). Shown are averages with SEM. Welch's t-test. (**B**) Experimental design is shown in right. A primary tumour induced the liver-to-lung relocation of HepELs (black-bordered). Red circle indicates light exposure area, and in this area, $CD45^{+}$ cells received photoconversion . The data used for this calculation (%) are displayed in Fig. 2C.

Appendix Fig. S6

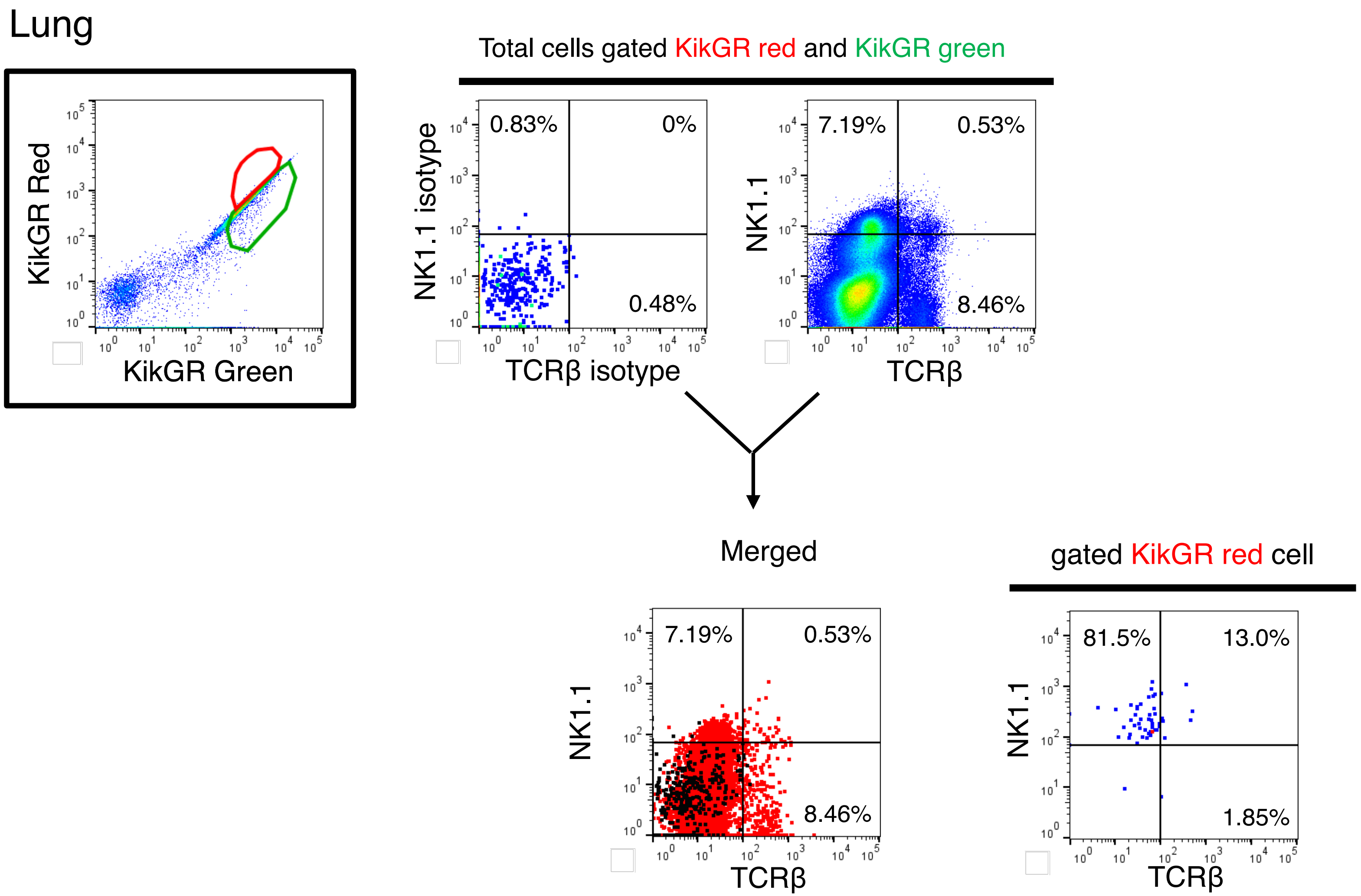

Gating strategy for lung cells that contained the relocated HepELs, which were photoconverted in liver in a TCM-stimulated KikGR mouse (gated in red polygonal region). Total cells gated KikGR red and KikGR green cell were separated using anti-NK1.1 and anti-TCRβ antibodies based on each isotype antibody. Percent is shown for total cells gated KikGR red and KikGR green cell (Merged). The gated KikGR red cells contained NK1.1^{+}TCRβ^{-} and NK1.1^{+}TCRβ dim but not NK1.1^{-}TCRβ^{+} T cells. Percent is shown for gated KikGR red cells (lower right).

Appendix Fig. S7

B220⁺CD11c⁺NK1.1⁺ cells

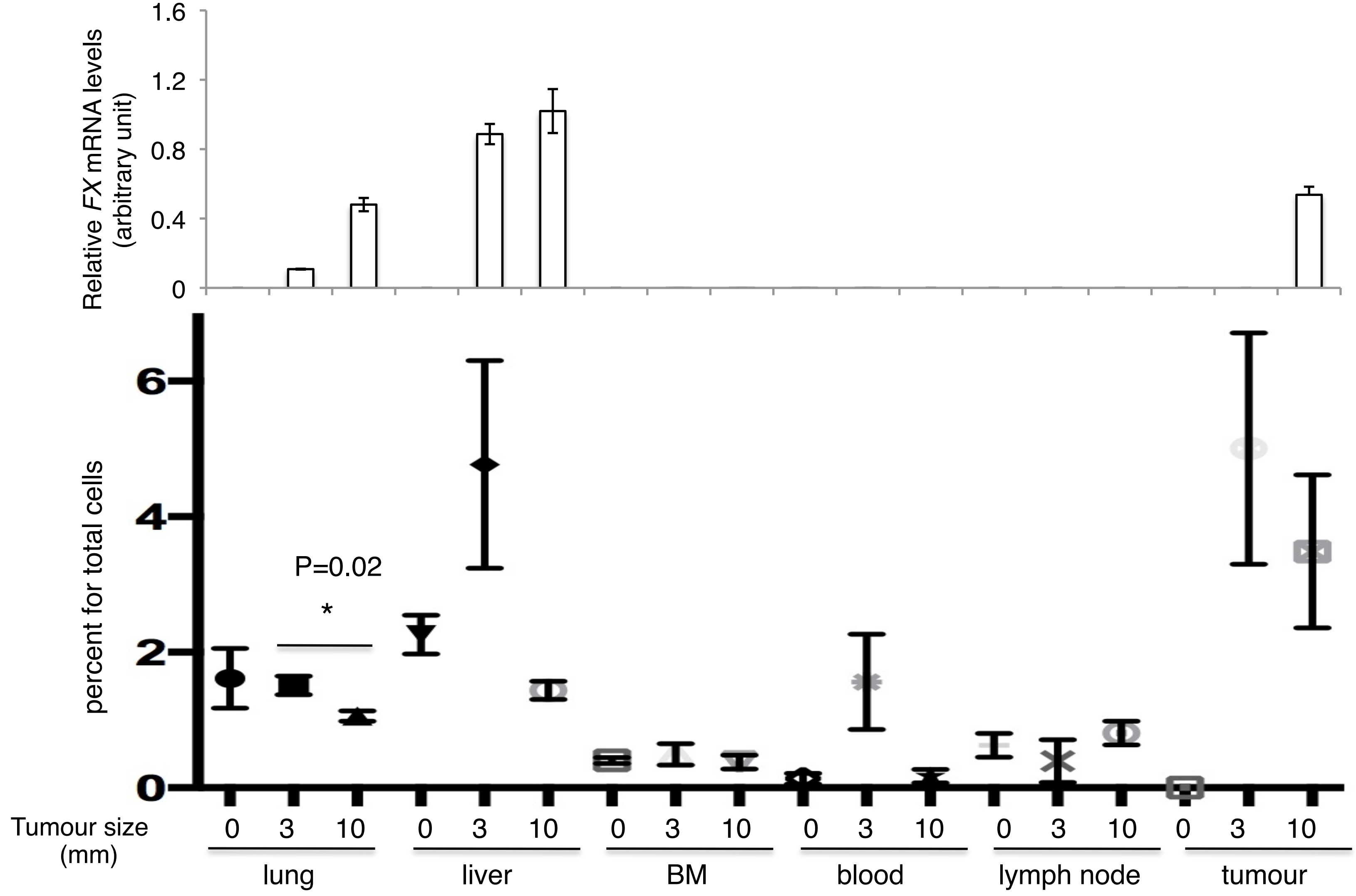

Tracing of B220$^+$CD11c$^+$NK1.1$^+$ cells in various organs such as lung, liver, bone marrow, peripheral blood, lymph node and primary tumours during the progression of tumour. The detection timing point is 0 mm in diameter of tumour (2 days), 3mm (7 days), and 10mm (14 days) after implantation of tumour cells. FX expression (upper) and flow cytometric analyses of B220$^+$CD11c$^+$NK1.1$^+$ cells (lower). N=4

Appendix Fig. S8

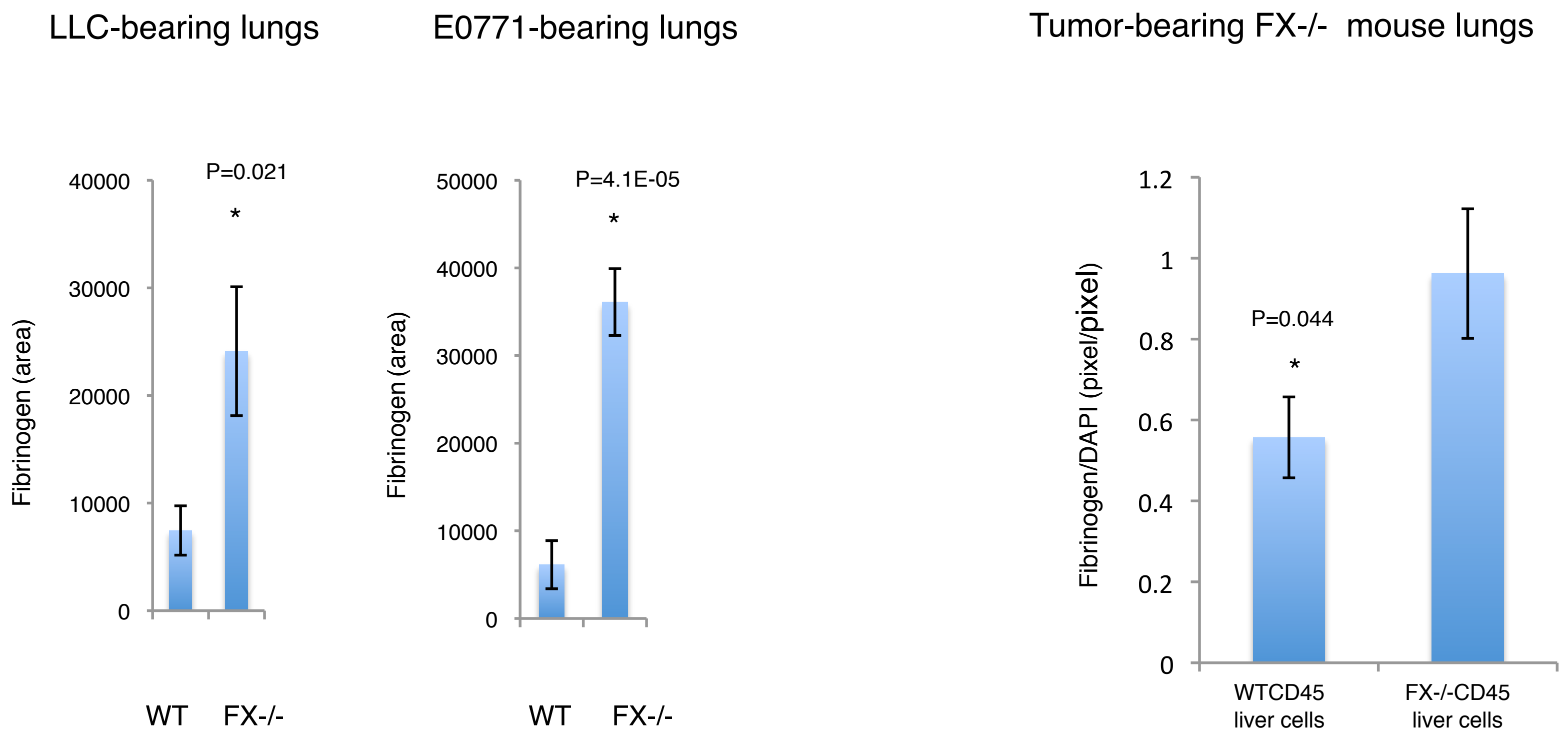

Quantification of fibrinogen deposition area in tumor-bearing wild-type (WT) and $FX^{-/-}$ mouse lungs (tumor-size matched analysis). N = 4, left.

Elimination of lung fibrinogen by liver $CD45^{+}$ cells derived from WT or FX-/- in tumor-bearing FX-/- mice. Quantification of fibrinogen area normalized by DAPI (lower). N = 4, right.

Appendix Fig. S9

Tumour-bearing FX-/- mouse lungs

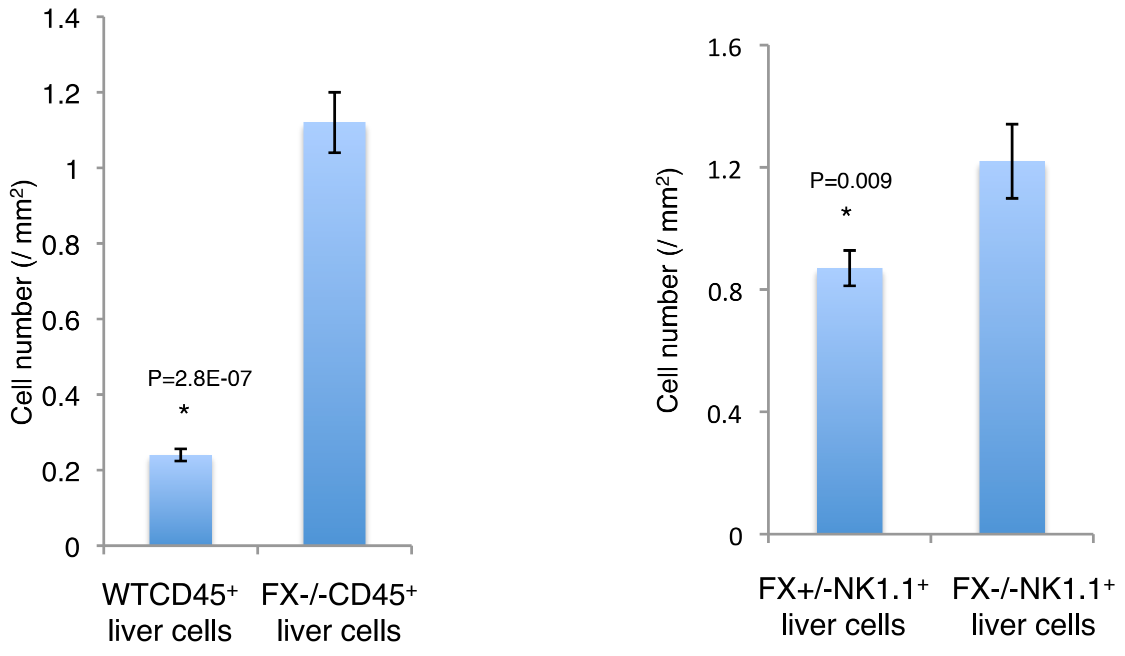

Metastatic tumor cells in tumor-bearing FX-/- mouse lungs that were treated with wild-type, FX+/- or FX-/- HepELs. Sorted cells were obtained with $CD45^+$ microbeads (left, N=5) and $NK1.1^+$ microbeads (right, N=3)

Appendix Table S1

		Array data<br>LLC-bearing	Fold change of tumour-bearing / no tumour-bearing<br>B16-bearing	 mean		
Arg1	GI:158966684	7.4	4.6	6	arginase, liver	
		5.5	6.2	5.85		BG060788
Cspg2	GI:197333875	5.5	5.5	5.5	versican	
	GI:12854022	5.6	5.2	5.4		BM251152
Spp1	GI:323668331	7.5	2.1	4.8	secreted phosphoprotein 1	
	GI:13435848	4.9	4.5	4.7		BC004774
		4.7	4.5	4.6		BG066773
		4.9	4.2	4.55		BB747681
	GI:12838759	5.3	3.8	4.55		AK005925
Cdx4	GI:145966782	4.7	4.1	4.4	caudal type homeobox 4	
Adducin2	GI:6851281	3.9	4	3.95	adducin2	
Scya2	GI:6531370	4	3.7	3.85	monocyte chemoattractant protein (MCP)-1	
		4.9	2.8	3.85		BI106821
Cldn13	GI:302564684	4.6	3.1	3.85	claudin 13	
		3.6	4.1	3.85		BB323723
		3.4	4.2	3.8		AW123502
	GI:12856357	3.8	3.6	3.7		AK017223
		4.7	2.3	3.5		AV326497
		3.4	3.4	3.4		BB667300
		3.2	3.5	3.35		BG071058
Slc7a8	GI:8394324	3.3	3.3	3.3	solute carrier family 7	
		3	3.5	3.25		AA185889
Mt1	GI:218931157	3.4	3	3.2	metallothionein 1	
	GI:12852290	3.4	3	3.2		AK014439
		3.4	2.9	3.15		AW108268
		2.8	3.3	3.05		AV251542
TfR	GI:54914	3	3	3	transferrin receptor	
F10	GI:334724423	2.8	3.2	3	coagulation factor X	
Rnase4	GI:13542727	3.1	2.8	2.95	ribonuclease, RNase A family 4	
		2.9	3	2.95		AI415298
		2.9	2.8	2.85		BB147418
		2.9	2.8	2.85		BB451404
		2.8	2.9	2.85		BB712040
ATF5-beta	GI:14150812	3.4	2.2	2.8	activating transcription factor 5-beta	
		3	2.6	2.8		AK015619
acrosin	GI:201009	2.6	2.8	2.7	acrosin	
		3.1	2.3	2.7		BB024472
		2.8	2.6	2.7		BB140565
		2.7	2.7	2.7		BF303544
		3	2.2	2.6		BB754834
	GI:12844551	2.8	2.4	2.6		AK009636
Sh3d19	GI:7657563	2.9	2.2	2.55	SH3 domain protein D19	
Hr	GI:560879478	2.7	2.4	2.55	hairless	
		2.9	2.2	2.55		AV290575
	GI:12855447	2.5	2.4	2.45		AK016615
		2.2	2.6	2.4		AK016969
Arhe	GI:14290473	2.3	2.4	2.35	Rho family GTPase 3	
		2.3	2.4	2.35		AK017968
Ms4a6d	GI:119637843	2.5	2.1	2.3	membrane-spanning 4-domains, subfamily A, member 6D	
Hmox1	GI:195947362	2.1	2.4	2.25	heme oxygenase (decycling) 1	

A list of the top 50 genes that were significantly up-regulated in peripheral leukocytes in LLC-bearing or B16-bearing mice.
A comparison of the gene expression level between tumour-bearing- and non-tumour-bearing- mouse leukocytes shown as fold changes. Coagulation factor X (FX=F10) is shown as red.

Appendix Table S2

lung > liver			liver > lung		
Probe name	Gene symbol	Fold change	Probe name	Gene symbol	Fold change
1458504_at	Zc3h12d	6.192023	1455098_a_at	Vtn	-9.335761
1435447_at	Mip	6.1570253	1449193_at	Cd5l	-7.4024057
1416841_at	1110059E24Rik	5.757809	1443570_at	Cops3	-7.0647583
1447329_at		5.2908344	1445639_at	9130014G24Rik	-6.8324075
1431102_at	Cep350	5.209877	1449084_s_at	Sh3d19	-6.6454644
1436692_at		5.1653757	1454892_at	Pitpnb	-6.6453357
1421811_at	Thbs1(TSP)	5.141252	1417378_at	Cadm1	-6.5729446
1458730_at		5.0260763	1450779_at	Fabp7	-6.561568
1455796_x_at	Olfm1	5.004083	1422814_at	Aspm	-6.4956884
1438525_at	Mllt10	4.9534855	1422595_s_at	5730470L24Rik	-6.4695883
1420788_at	Klrg1	4.9360604	1445388_at	Cd226	-6.4644823
1419038_a_at	Csnk2a1	4.928009	1448680_at	LOC100046946 /// Serpina1c	-6.375888
1450700_at	Cdc42ep3	4.904225	1420064_s_at	Tktl1	-6.3682814
1442928_at		4.759984	1450624_at	Bhmt	-6.3638434
1420659_at	Slamf6	4.735423	1444263_at		-6.3079257
1444128_at	Arhgap26	4.727369	1459742_at		-6.277002
1443919_at	Prrt3	4.669039	1416931_at	Nif3l1	-6.2696247
1429997_at	4832441B07Rik	4.623043	1429921_at	9530068E07Rik	-6.250474
1427381_at	Irg1	4.59012	1452319_at	Zfp82	-6.191312
1457948_at	Gas7	4.5334888	1416225_at	Adh1	-6.1089277

Gene expression in photoconverted HepELs derived from TCM-stimulated- lungs and livers.
Top 20 up-regulated genes in HepELs that migrated from the liver to the lung compared with those that stayed in the liver in TCM-stimulating mice (left). Top 20 up-regulated genes in non-migrating HepELs in the liver compared to HepELs in lungs in the same mice (right). To perform this microarray analysis, complementary DNAs obtained from five mice were combined before the hybridization process. The fold changes of mRNA levels in liver versus lung were shown. The fibrinogen-binding molecules have been reported as red.
