# Supplementary material for: Hepato‐entrained B220+ CD11c+ NK1.1+ cells regulate pre‐metastatic niche formation in the lung
Source: EMBO Mol Med. 2018 Jun 21;10(7):e8643. doi: 10.15252/emmm.201708643 (PMC6034134; doi:10.15252/emmm.201708643)
Supplement: Supplementary file 2 — Expanded View Figures PDF [file EMMM-10-e8643-s002.pdf]

Expanded View Figures

Figure EV1. Flow cytometric analyses of moving HepELs in a KikGR mouse lungs.

A–E Representative flow cytometric analysis in lungs 72 h after liver exposure to violet light. Cells obtained from mouse with no light exposure gave KikGR red versus KikGR green dot plot (A). Those with light exposure (B, D) exhibited photoconverted cells in a red polygonal region. Cells in the gated region were further analysed by using CD4, CD8, CD45, CD11c, CD11b and B220 antibodies (C, E). Percent is shown for total cells in (A, B and D) and for gated cells in (C and E). conCM (control) and TCM were injected (3 times, every 2 days). Comparison of isotype control-Ab and specific-Ab are also shown (Appendix Figs S3 and S4).

© 2018 The Authors

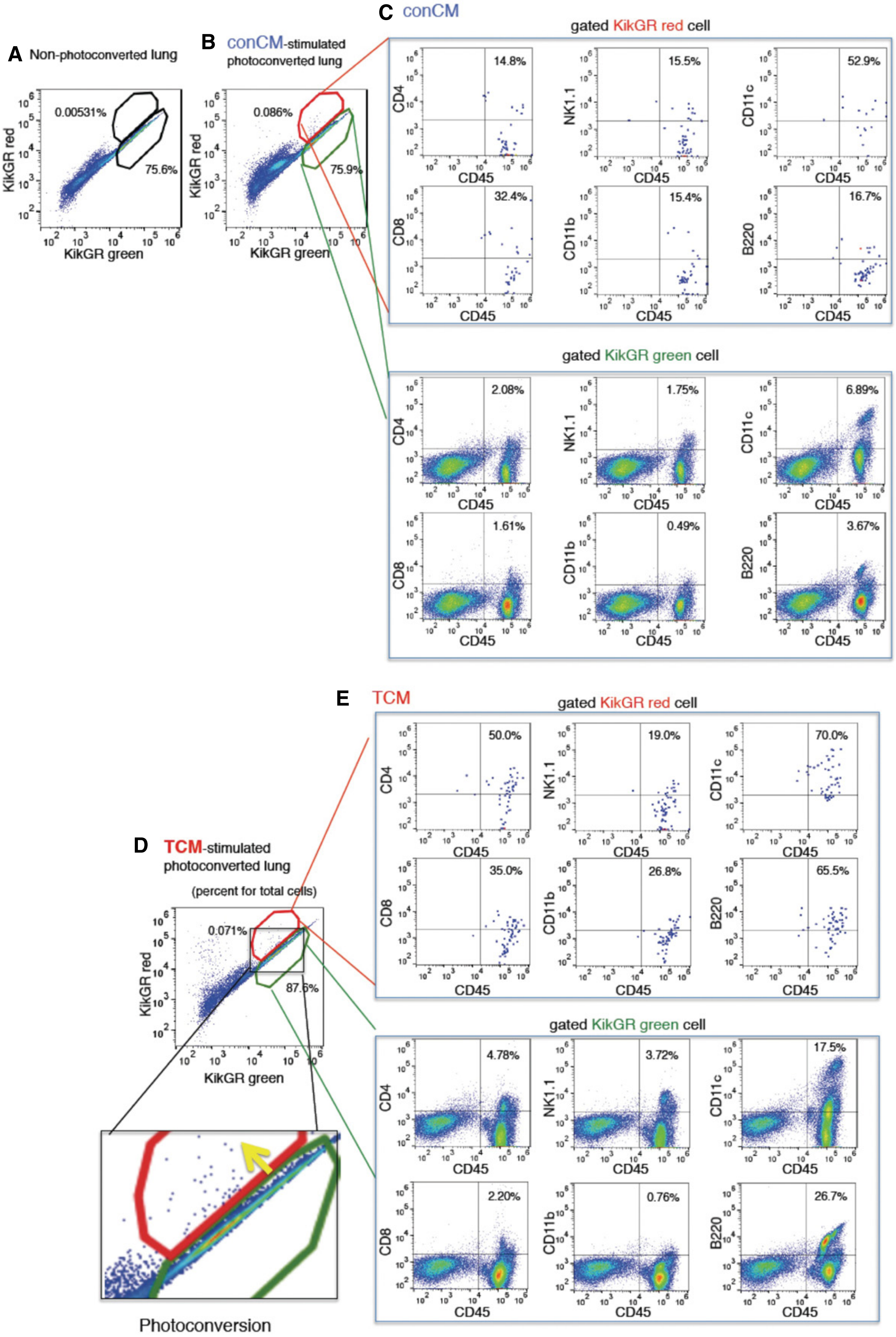
A Non-photoconverted lung
0.00531%
75.6%
KikGR red
KikGR green
B conCM-stimulated photoconverted lung
0.086%
75.9%
C conCM
gated KikGR red cell
14.8%
15.5%
52.9%
32.4%
15.4%
16.7%
CD4
NK1.1
CD11c
CD8
CD11b
B220
CD45
gated KikGR green cell
2.08%
1.75%
6.89%
1.61%
0.49%
3.67%
D TCM-stimulated photoconverted lung
(percent for total cells)
0.071%
87.6%
Photoconversion
E TCM
gated KikGR red cell
50.0%
19.0%
70.0%
35.0%
26.8%
65.5%
gated KikGR green cell
4.78%
3.72%
17.5%
2.20%
0.76%
26.7%

Figure EV1.

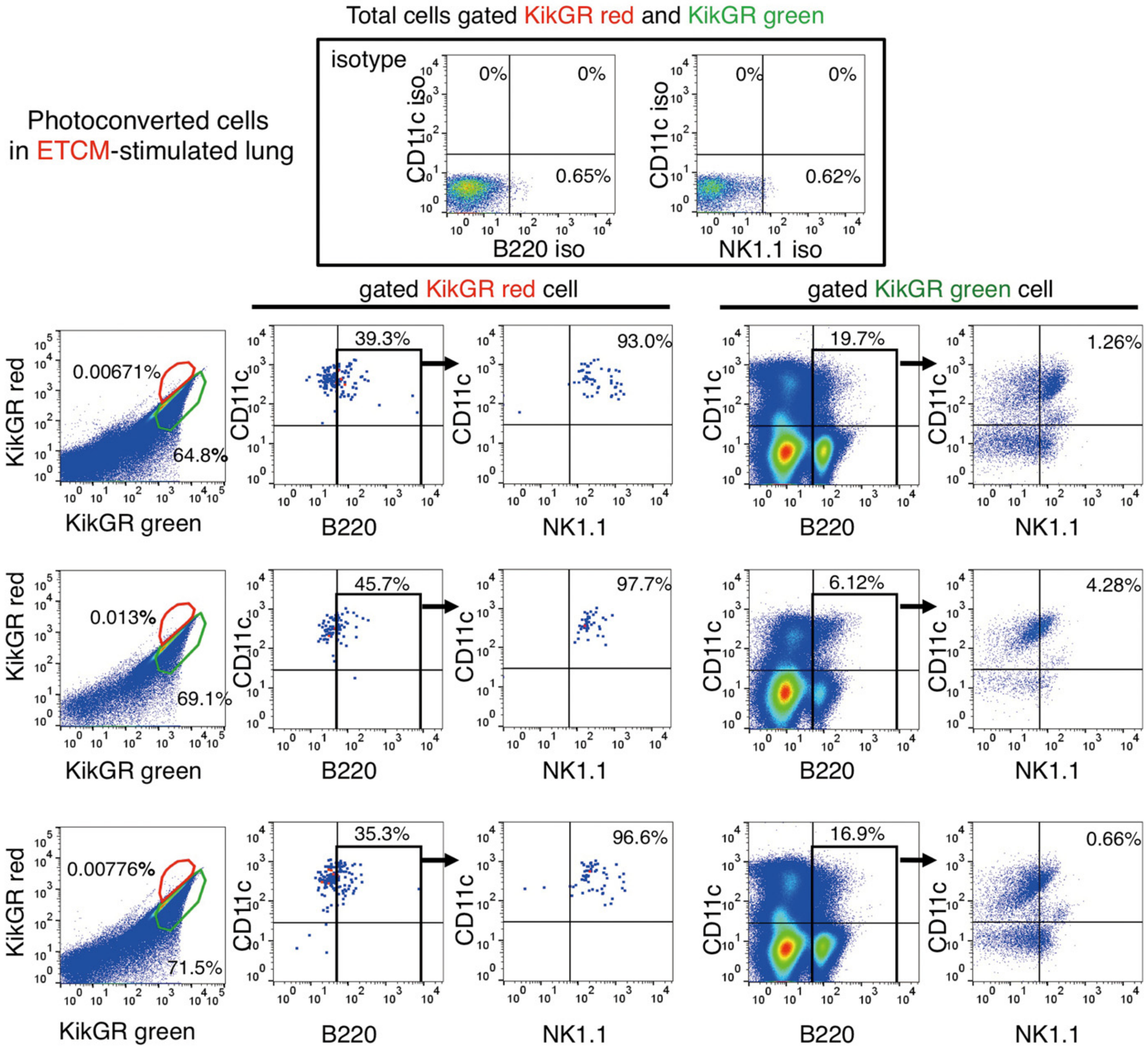

Figure EV2. Representative three independent flow cytometric analyses related to Fig 3B.

The movement of B220^{+}CD11c^{+}NK1.1^{+} cells in lungs that were photoconverted in liver of a E0771-TCM (ETCM)-stimulated KikGR mouse. Percent is shown for total cells in the upper panel (total cells gated KikGR red and KikGR green) and for gated cells in the lower panels.

© 2018 The Authors

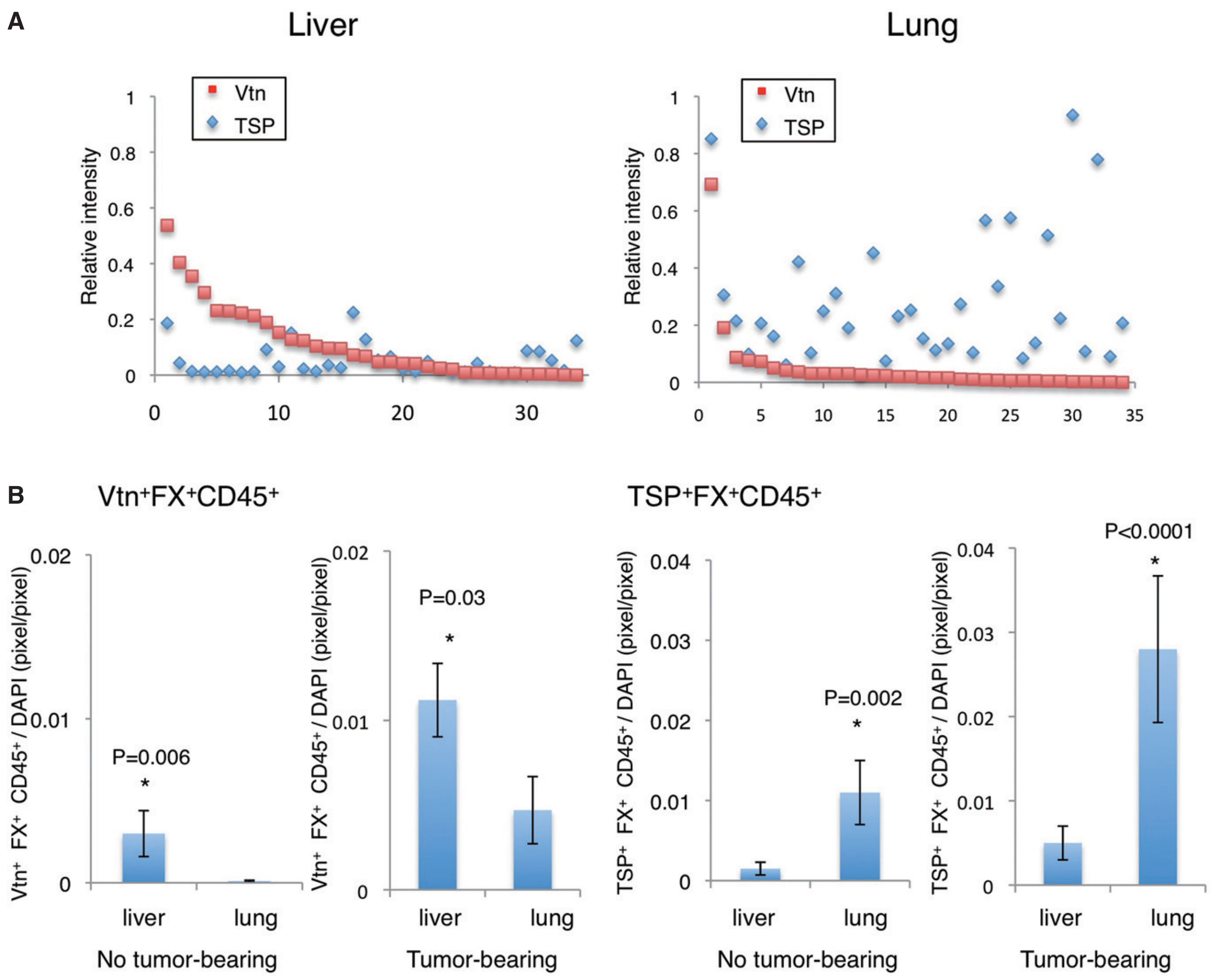

Figure EV3. Differential Vtn and TSP expressions in FX⁺CD45⁺ cells in the liver and lungs of cancer patients.

A Different patterns of Vtn and TSP expressions in liver CD45⁺ cells and lung CD45⁺ cells in cancer patients. Relative expression levels of Vtn and TSP in FX⁺CD45⁺ cells as determined by immunostaining. The perpendicular axis represents the signal intensities of Vtn (red squares) and TSP (blue diamonds) in FX⁺CD45⁺ cells in sample sets of liver (left panel) and lung (right panel) cells derived from breast cancer-bearing patients. The horizontal axis represents the sample numbers. Each individual sample is given one number (N = 7 patients, 4–5 samples/patient, total 34 samples). Each value was normalized by DAPI immunostaining.

B Immunohistochemical quantitative analysis of Vtn⁺FX⁺CD45⁺ cells and TSP⁺FX⁺CD45⁺ cells in the liver and lungs. Tumour-free human samples were also shown (N = 12, 70 samples). Shown are averages with SEM. Welch's t-test.

© 2018 The Authors
