# Supplementary figures and images for: Hepato‐entrained B220+ CD11c+ NK1.1+ cells regulate pre‐metastatic niche formation in the lung
Source: EMBO Mol Med. 2018 Jun 21;10(7):e8643. doi: 10.15252/emmm.201708643 (PMC6034134; doi:10.15252/emmm.201708643)

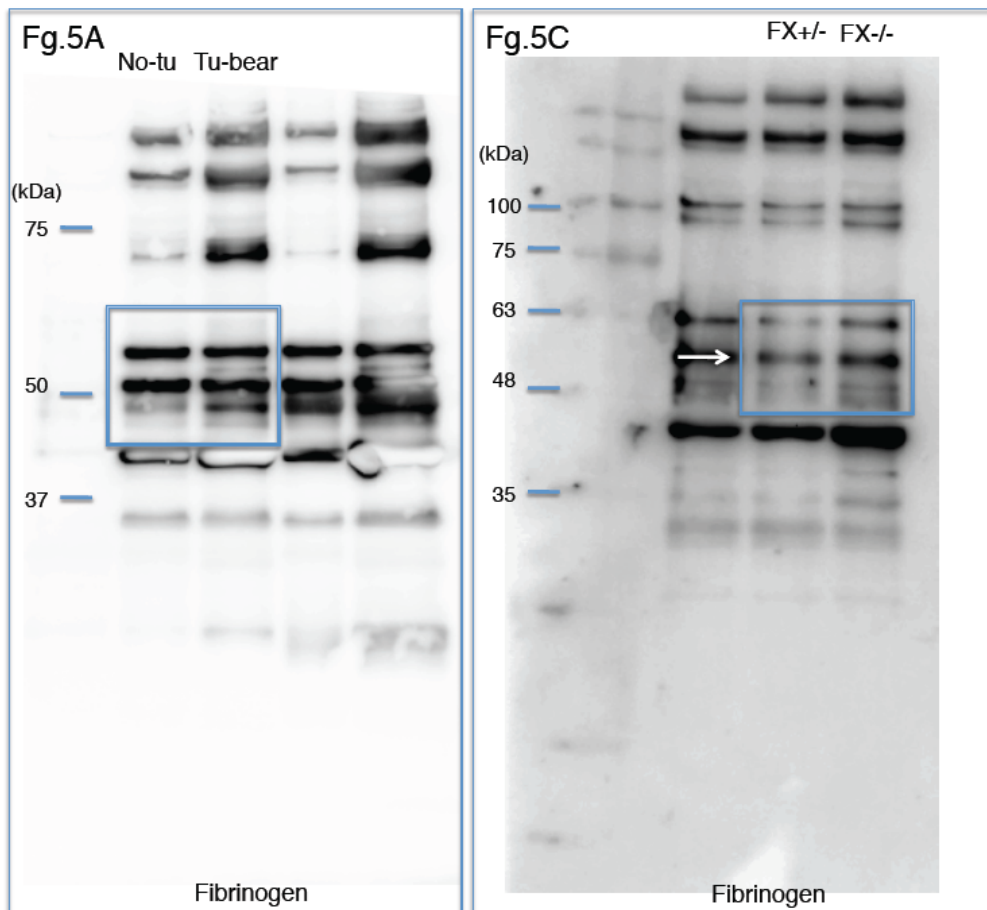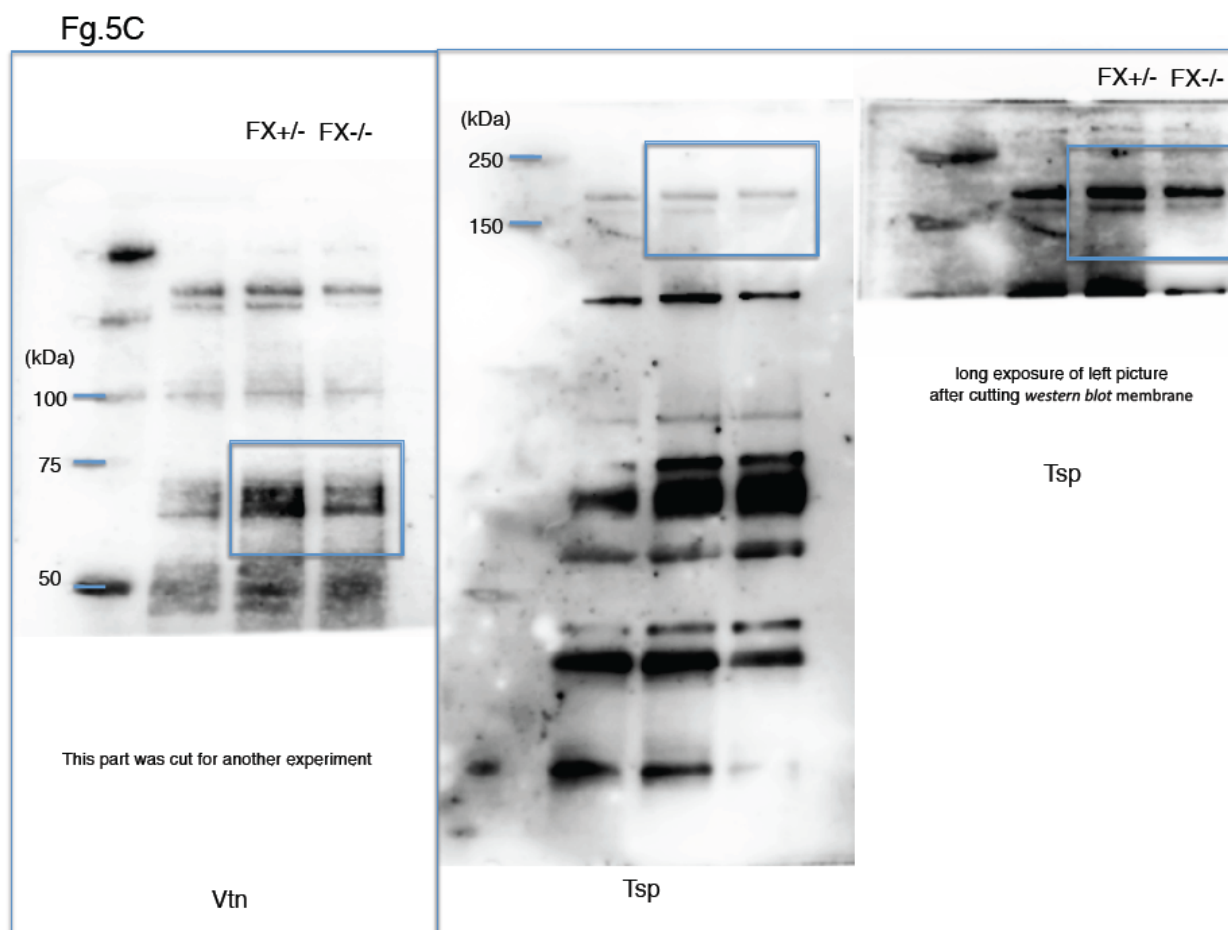

Supplement: Supplementary file 4 — Source Data for Figure 5 [file EMMM-10-e8643-s003.pdf]
